# Newly Diagnosed Systemic Lupus Erythematosus in a 14-Year-Old Female

**DOI:** 10.7759/cureus.83296

**Published:** 2025-05-01

**Authors:** Hannah Nix, Erin S Reid, Kate E Gargiula, Ashley George, Amanda Gold

**Affiliations:** 1 Internal Medicine, Alabama College of Osteopathic Medicine, Dothan, USA; 2 Pediatrics, Gulf Shores Pediatrics, Gulf Shores, USA

**Keywords:** antinuclear antibodies, autoimmune disorders, biologic treatment, jsle, malar rash

## Abstract

Juvenile-onset systemic lupus erythematosus (jSLE) is a rare, life-threatening autoimmune disease that can be seen in the pediatric population. jSLE patients are more prone to highly aggressive forms of this condition when compared to adult-onset counterparts. This makes early recognition and adequate immunosuppressive therapies key in preventing end-organ damage. SLE is classically managed clinically with antimalarials and glucocorticoids, but injectable biologic therapies are slowly becoming part of mainstream care. Our case report highlights a 14-year-old female who presents with a persistent facial rash, headaches, and weight loss following treatment for Influenza B.

## Introduction

Juvenile-onset systemic lupus erythematosus (jSLE) is a rare autoimmune disease that presents before the age of 18. It is an extremely inflammatory disease that can have multi-system organ involvement [1]. The pathophysiology of systemic lupus erythematosus (SLE) is complicated and poorly understood but advances in research have shed some light on the autoantibodies and genetic disposition that are associated with this disease process.

B cells are the central figure of SLE as they are the cell type responsible for the secretion of antibodies and autoantibodies. While there are almost a dozen autoantibodies that can be implicated in SLE, the most specific for diagnosis are anti-dsDNA and anti-Smith antibodies. Additionally, antinuclear antibody (ANA) is sensitive but not specific for SLE and is commonly used in screening and diagnosis. In jSLE patients, anti-dsDNA antibodies are detected in 70-98% of cases [2]. Anti-double-stranded DNA (anti-dsDNA) antibodies are highly specific for SLE and are not typically associated with other autoimmune diseases, making them a crucial component in the diagnostic evaluation of lupus. Various studies have also shown that titers of anti-dsDNA antibodies rise during flares of SLE, particularly in cases of lupus nephritis [3]. ANA tests can be highly elevated when underlying autoimmune pathology is taking place, making them a common first-line test when autoimmunity is suspected. Elevated ANA can also be seen in infections, malignancy, and dozens of other autoimmune disorders, so alone they are not considered specific diagnostic criteria for SLE.

Historically, B cell-activating factor (BAFF), also known as B lymphocyte stimulator protein, is elevated in patients with SLE. In lupus patients, BAFF works by promoting the survival of autoreactive B cells, thereby increasing the amount of autoantibodies attacking the body [4].

The presentation of jSLE in the pediatric population is highly variable making it a complex diagnosis and can further complicate the treatment process. The typical signs and symptoms of adult-onset SLE include: fatigue, weight loss, fever, generalized arthralgias and the characteristic malar rash [5]. In jSLE, the symptoms are typically more severe and usually display neuropsychiatric and hematological abnormalities [6]. Most of these symptoms are seen in a wide variety of disease processes, which makes the differential diagnoses extensive. For this reason, the American College of Rheumatology and European League Against Rheumatism (ACR/EULAR) created criteria that make identifying SLE in a patient more straightforward [5]. The criteria start with analyzing a patient’s ANA-titer to determine if it meets the entry criterion. If the patient’s titer levels qualify for the entry criterion, then their clinical manifestations and diagnostic work-up are graded based upon the weight given by the classification. The criteria take into account multiple systems including hematological, renal, mucocutaneous, neuropsychiatric, and many others [7]. The weight of each applicable criterion is added together to give the patient’s total score. If the score is 10 or greater, then it is classified as SLE [7]. This classification system can also be used in the diagnosis of jSLE with the same cutoff score of 10 or greater. When implemented with our case, the patient’s calculated score was >24.

## Case presentation

A 14-year-old female with an unremarkable past medical history presented to the pediatric clinic with the chief complaint of a fever. She had the fever for less than twenty-four hours with a maximum temperature of 101.5 F. She also complained of a rash, joint pain, and periodic headaches. The rash had been present for six weeks, worsened with activity, and was located over the bilateral buccal regions. She describes transient arthralgia, located in the right ankle and bilateral knees, that resolved after one day. The patient denied any myalgias, joint swelling, muscle weakness, changes in daily activities, energy decrease, weight loss, appetite changes, dysuria, changes in urinary or bowel habits, nausea, vomiting, night sweats, any potential tuberculosis exposure, or recent travel.

Vital signs and growth chart percentiles when presenting to the clinic are shown in Tables 1, 2.

**Table 1:** Vital signs on presentation to clinic

Vital Signs	Values	Normal Range
Blood Pressure	116/74	90-120/50-80 mmHg
Heart Rate	65	60-100 beats per minute
Respiratory Rate	17	12-20 breaths per minute
Temperature	99.4 F	97.6-99.6 F
Pulse Ox	99%	95-100%
Body Mass Index (BMI)	21.41	18.5-25 kg/m^2^
Weight	144 lbs	84-160 lbs

**Table 2:** Patient's growth chart percentiles on presentation to clinic

Type of Growth Chart	Percentile	Normal Range
Weight	86	5-85 lbs
Height	>97	3-97 in
Body Mass Index (BMI)	68	5-85 kg/m^2^

A significant finding was a nine-pound weight loss from her last visit, with a reweigh to confirm. On physical exam, remarkable findings included a faintly erythematous, non-pruritic, smooth macular, circular rash without a well-demarcated border, scales, bumps, or nodular lesions on the face in a malar distribution. She also had bilateral cervical chain lymphadenopathy, with the right side being greater than the left. Because of these remarkable findings, a full workup including complete blood count with differential, ferritin, C-reactive protein, erythrocyte sedimentation rate, immunoglobulin G and immunoglobulin A levels, and ANA w/Reflex titer was ordered. Labs are shown in Table 3 and were significant for leukopenia, decreased red blood cells, hemoglobin, and hematocrit. These findings, along with increased ferritin and mean corpuscular volume, mean corpuscular hemoglobin, mean corpuscular hemoglobin concentration, and red cell distribution width within normal limits, confirm a normocytic anemia most likely due to anemia of chronic disease.

**Table 3:** Patient's significant findings from CBC with differential and ferritin levels

	Patient Value	Normal Range
White Blood Cells (WBC)	3.4	6.3-10 k cells/mcL
Red Blood Cells (RBC)	3.92	4.20-5.40 million cells/mcL
Hemoglobin	11.2	11.6-14.8 g/dL
Hematocrit	33.9	34.0-44.0%
Mean Corpuscular Volume	86.6	82.0-98.0 fL
Mean Corpuscular Hemoglobin	28.7	25.0-29.0 pg/cell
Mean Corpuscular Hemoglobin Concentration	33.1	32.0-36.0 g/dL
Red Blood Cell Distribution Width	12.9	11.5-15.1 fL
Ferritin	74	26-388 ng/mL

Other significant lab findings, shown in Table 4, included elevated total protein, globulin, ESR, and CRP. She also had remarkable lab findings that included decreased A/G, low C3 and C4 complement levels, elevated IgG and IgA, elevated anti-DNA antibodies, and a positive ANA screen.

**Table 4:** Other significant lab findings in the initial workup

	Patient Value	Normal Range
Total Protein	9.1	6.4 - 8.2 g/dL
Globulin	5.0	2.8 - 3.2 g/dL
Erythrocyte Sedimentation Rate (ESR)	46	0 - 20 mm/hr
C-Reactive Protein (CRP)	0.7	0 - 2.0 mg/L
Albumin/Globulin (A/G)	0.8	1 - 2 g/dL
C3 complement	65	82 - 175 mg/dL
C4 complement	3	13 - 46 mg/dL
Immunoglobulin G (IgG)	1929	500 - 1590 mg/dL
Immunoglobulin A (IgA)	323	36 - 220 mg/dL
Anti-DNA antibodies	522	= 4 IU/mL
Antinuclear antibody (ANA) screen	Positive	Negative

Since the ANA screen was positive, an ANA titer was done, as shown in Table 5. It was elevated with a nuclear, speckled pattern associated with conditions such as mixed connective tissue disease, systemic lupus erythematosus, Sjogren’s syndrome, dermatomyositis, or systemic sclerosis/polymyositis.

**Table 5:** Other significant findings during the initial workup

	Patient Value	Normal Value
Antinuclear antibody (ANA) titer	1:1280	<1:40 IU/mL

Because of her presentation and significant laboratory findings, she was referred to rheumatology. Once seen at rheumatology, she reported new additional symptoms such as fatigue, hair loss, generalized morning stiffness that lasts for around an hour, continuous headache, and an erythematous eczematous lesion on her left eyelid. These findings, along with her labs, supported the diagnosis of systemic lupus erythematosus. She was prescribed Plaquenil 200 mg daily, Benlysta 200 mg subQ injection once every seven days, CellCept 250 mg twice daily for five days followed by 500 mg twice daily, prednisone 40 mg daily for seven days, then 30 mg daily for five days, then 20 mg daily with her steroid dose being tapered at follow-up visits. She was also prescribed Pepcid 20 mg twice daily before meals while taking prednisone.

## Discussion

**Figure 1:**
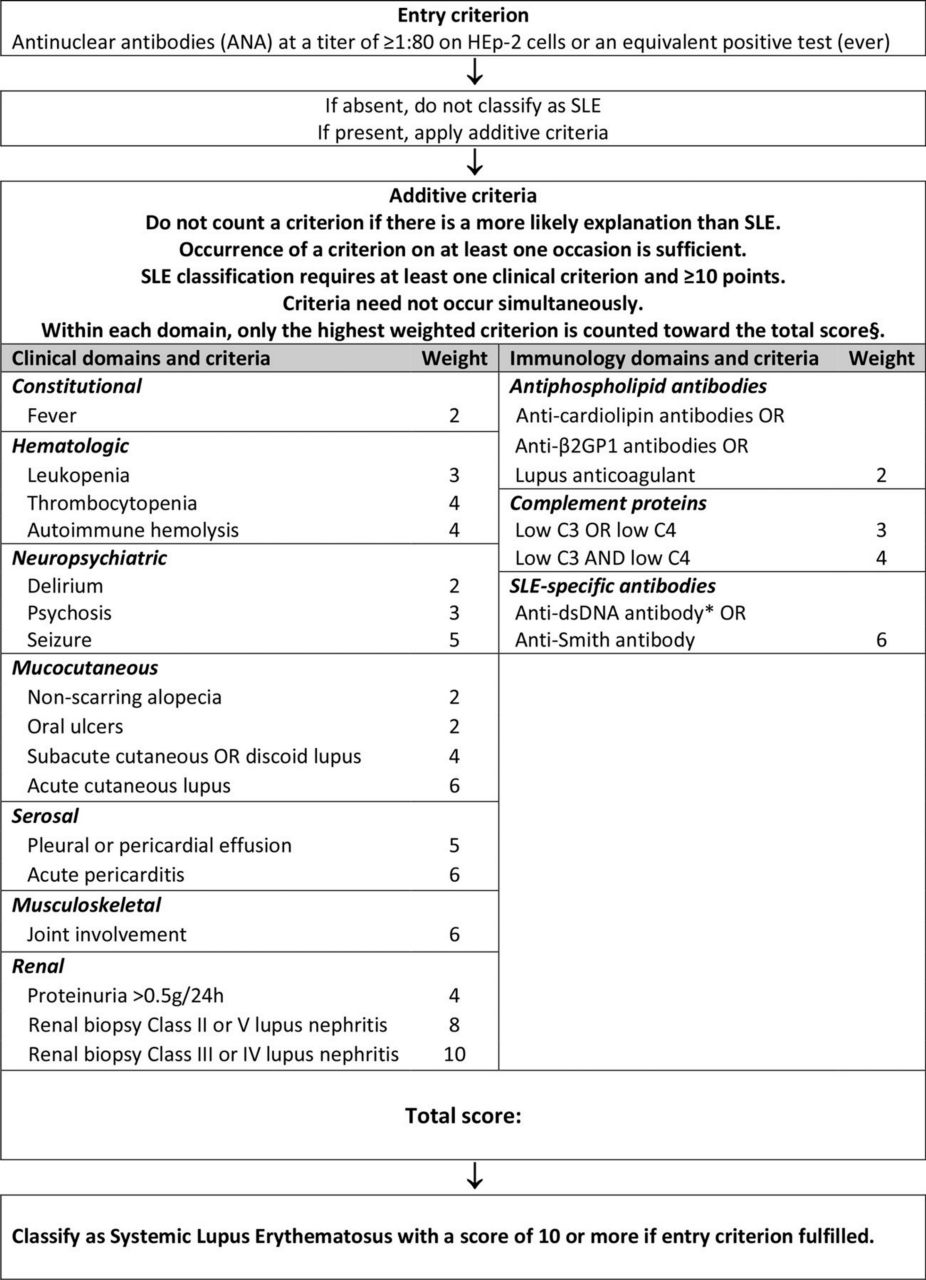
American College of Rheumatology and European League Against Rheumatism (ACR/EULAR) criteria for diagnosing SLE

The diagnosis of jSLE can be a challenging task for any physician due to the rarity of its presence in a pediatric population. jSLE has an incidence of 0.3-0.9 per 100,000 children per year and a peak age of onset at 12.6 years [8]. Typical presentations include mucocutaneous manifestations, fevers, fatigue, weight loss, renal, cardiopulmonary and rheumatological involvement. The wide breadth of systems that can be involved in diagnosis allows for multiple possible combinations of presenting symptoms as well as endless differential possibilities. The EULAR/ACR criteria for the classification of systemic lupus erythematosus, shown in Figure 1, are a well-accepted criterion system for the diagnosis of SLE in adults with a score >10 leading to diagnosis of SLE. Our patient received a calculated score >24 [9].

Following diagnosis of jSLE, initiating prompt and well-tolerated immunosuppressive therapies is the hallmark of increasing quality of life and decreasing sequelae of life-threatening conditions such as lupus nephritis, diffuse alveolar hemorrhage, stroke, etc. Traditional therapies include hydroxychloroquine, mycophenolate mofetil, azathioprine, methotrexate, and prednisone. New therapies, specifically biological medications, have begun making their way into the mainstream as an adjunct treatment for SLE. Our patient was placed on Belimumab in addition to traditional treatment strategies utilizing hydroxychloroquine, mycophenolate mofetil, and prednisone. Belimumab (Benlysta) is a human monoclonal antibody that can be taken as an at-home subcutaneous injection once every week. Belimumab works by binding the B lymphocyte stimulator protein also known as B cell activating factor; this action then inhibits the survival of autoreactive B cells and reduces the differentiation of B cells into immunoglobulin-secreting plasma cells [4]. B cells play a critical role in the development and severity of SLE by producing excessive autoantibodies that target the body's DNA. In patients with lupus who were positive for anti-double-stranded DNA, treatment with BENLYSTA resulted in a 41% reduction in anti-double-stranded DNA antibody levels over 52 weeks. Additionally, increases in complement proteins C3 and C4 were also observed at week 52 in patients with low complement levels at baseline [4]. The addition of Belimumab to a treatment regimen not only has the possibility to better control SLE but also can lower the dose of oral corticosteroids needed [10]. In a juvenile population, limiting the dose and frequency of oral corticosteroid can have many beneficial impacts on their future quality of life due to decreasing long-term steroid adverse events such as osteoporosis, weight gain, hypertension, hyperglycemia, glaucoma and cataracts, early mortality, etc.

jSLE patients should be closely monitored by their healthcare team due to variability of the disease course. Annual screenings, such as eye exams and thorough skin exams, should be recommended to new patients due to treatment regimen side effects. Hydroxychloroquine has the potential to cause retinal toxicity and azathioprine can lead to an increased chance of skin cancer [11]. Additionally, annual labs checking hepatic function and renal function are recommended. Patients receiving glucocorticoid therapy as a primary treatment may benefit from early initiation of bone mineral density assessments, such as DEXA scans. Preventative lifestyle modifications - including regular use of sunscreen, abstaining from alcohol, and engaging in moderate-intensity weight-bearing exercise - can also help mitigate long-term side effects [11]. Our patient was counseled on these recommended screening and lifestyle measures. In addition, close follow-up was advised to monitor disease activity and ensure symptom control until a stable treatment regimen was achieved.

## Conclusions

Juvenile systemic lupus erythematosus is a multifactorial and extremely inflammatory autoimmune disease that presents in adolescence. It can affect multiple organ systems and cause irreversible damage to the body. Due to the highly variable nature of the disease course and symptoms, it can be challenging to promptly diagnose jSLE. For this reason, when clinical suspicion arises, a comprehensive diagnostic work-up should be performed. Prompt treatment should be started once the diagnosis is made. The use of biological agents alongside conventional therapeutic regimens may improve outcomes in select patients. However, jSLE still lacks well-defined treatment algorithms, particularly regarding the initiation of biological therapies in newly diagnosed cases. Consequently, long-term clinical studies focused on the management of jSLE are critically needed and remain a priority within rheumatology subspecialties.
